# Microwave-assisted biodiesel production using bio-waste catalyst and process optimization using response surface methodology and kinetic study

**DOI:** 10.1038/s41598-023-29883-4

**Published:** 2023-02-13

**Authors:** Rhithuparna Devasan, Joseph V. L. Ruatpuia, Shiva Prasad Gouda, Pravin Kodgire, Sanjay Basumatary, Gopinath Halder, Samuel Lalthazuala Rokhum

**Affiliations:** 1 Department of Chemistry, National Institute of Technology, Silchar, Assam 788010 India; 2 Chemical Engineering Department, Pandit Deendayal Energy University, Gandhinagar, Gujarat 382426 India; 3 Center for Biofuel and Bioenergy Studies, Pandit Deendayal Energy University, Gandhinagar, 382426 India; 4 Department of Chemistry, Bodoland University, Kokrajhar, Assam 783370 India; 5 Department of Chemical Engineering, National Institute of Technology Durgapur, Durgapur, West Bengal 713209 India

**Keywords:** Chemistry, Energy science and technology

## Abstract

Providing sufficient energy supply and reducing the effects of global warming are serious challenges in the present decades. In recent years, biodiesel has been viewed as an alternative to exhaustible fossil fuels and can potentially reduce global warming. Here we report for the first time the production of biodiesel from oleic acid (OA) as a test substrate using porous sulfonic acid functionalized banana peel waste as a heterogeneous catalyst under microwave irradiation. The morphology and chemical composition of the catalyst was investigated using Powder X-ray diffraction (PXRD) analysis, Fourier transform infrared (FTIR) spectroscopy, Thermogravimetric analysis (TGA), Transmission electron microscopy (TEM), and Scanning electron microscopy- Energy dispersive X-ray spectroscopy (SEM–EDX). The SEM–EDX analysis of the catalyst revealed the presence of sulfur in 4.62 wt% amounting to 1.4437 mmol g^−1^ sulfonic acids, which is accorded to the high acidity of the reported catalyst. Using response surface methodology (RSM), through a central composite design (CCD) approach, 97.9 ± 0.7% biodiesel yield was observed under the optimized reaction conditions (methanol to OA molar ratio of 20:1, the temperature of 80 °C, catalyst loading of 8 wt% for 55 min). The catalyst showed excellent stability on repeated reuse and can be recycled at least 5 times without much activity loss.

## Introduction

The demand for burning fossil fuels is growing day by day as a result of fast urbanization and population growth. These fuels will run out in time at the current consumption rate^1^. Researchers have been looking for ways to replace fossil fuels with renewable and environmentally benign energy sources due to the chronic need for energy and the inevitable depletion of crude oil reserves worldwide^2,3^. In addition, fossil fuel consumption results in severe environmental pollution by elevated atmospheric CO_2_ concentrations, thereby increasing global warming^4,5^. As a result, it is imperative to meet rising energy demands while simultaneously reducing CO_2_ emissions to prevent catastrophic global warming amicably and sustainably using renewable resources. Although few alternative energy resources can resolve future energy needs by reducing greenhouse gas emissions, because of the existing infrastructure, biodiesel, which is made up of long-chain fatty acid methyl esters (FAMEs)^6,7^, appears to be the most viable, readily implemented alternative to conventional diesel fuel^8–10^. Besides being sustainable, renewable^11^, nontoxic, biodegradable^12^, carbon–neutral, and safe to handle, biodiesel produces low emissions throughout its life cycle as an esterification product of vegetable oil and methanol^6,13,14^. As an additional benefit, normal diesel engines can operate on biodiesel without modifications^15^. Biodiesel can be made from various feedstocks such as soybean, canola, palm, and rapeseed oils^16–19^. The cost of edible oil-based raw materials for biodiesel synthesis accounts for 70–80% of the total cost, making biodiesel production more expensive than diesel^20^.

Several works are being done to reduce the cost of these raw materials. Lathiya et al.^21^ reported a sulfonated carbon catalyst from waste orange peel through carbonization followed by sulfonation treatment, Khan et al.^22^ targeted agro waste derived metal oxide, and Zhao et al.^23^ used pomelo peel biochar for biodiesel synthesis. Non-edible sources such as waste cooking oil (WCO) and *Jatropha curcas* have been identified as good substitutes^24^. They tend to contain higher free fatty acid (FFA) levels, which makes them less suitable for alkali-catalyzed processes. Substantial FFA present in the feedstock can adversely affect the performance of the alkaline catalyst, as they react with the catalyst, causing soap and water formation through the saponification process^25^. Moreover, this type of reaction complicates downstream product separation, which results in an extended production process that is more expensive to operate.

Hence, choosing a proper catalyst is especially important. Generally, acid-catalyzed esterification of FFAs is preferred since the catalyst is designed to tolerate high FFA levels in the feedstock. Sulfuric acid (H_2_SO_4_) is the most commonly used conventional catalyst. According to the study done by Aranda et al.^26^, H_2_SO_4_ exhibited higher esterification activity in palm fatty acids. The H_2_SO_4_ has a higher catalytic activity due to its ability to protonate the carboxylic moiety of fatty acids and create tetrahedral intermediates. It is more economical and practical to catalyze biodiesel production with heterogeneous than homogeneous catalysts^27^ because of its many advantages, including ease of regeneration, time-saving, reduced corrosiveness, safety, and cost. Mateo et al.^28^ reported a sulfonated carbon catalyst developed from agricultural wastes via a one-step process involving direct carbonization and sulfonation with H_2_SO_4_ as a reagent. Mendaros et al.^29^ also synthesized a solid carbon acid catalyst by direct sulfonation of cacao shell for the esterification of oleic acid. Shang et al.^30^ used agricultural byproduct peanut shell, Kumawat, and Rokhum^31^ used orange peel waste for simultaneous carbonization and sulfonation with H_2_SO_4_ as a reagent. Hassan et al.^32^ revealed that using H_2_SO_4_ for sulfonation is of low cost and provides high acidity to the carbonaceous Bentonite catalyst. Ngaosuwan et al.^33^ predicted that these sulfonated-carbon catalysts in industries for catalytic reactions like esterification, transesterification, nitration, and cellulose hydrolysis could soon replace homogeneous H_2_SO_4_ catalyst.

Response surface methodology (RSM) is used to evaluate the impact of two or more independent factors on a complicated set of dependent variables. It uses numerous regression and correlation analyses to reduce the number of experiments required to gather adequate data for a statistically valid result. Central composite design (CCD) is one of the most popular RSM response design experiments. This method has already been utilized to improve biodiesel yield from a wide range of feedstocks and catalysts^27^. Doehlert Design (DD), Box-Behnken Design (BBD), and full factorial design are the types of design used for the RSM study, including CCD^34^. However, because of the benefit of optimizing multifactor problems with the minimum number of experimental runs, CCD is extensively employed for optimization procedures. The CCD model permits two-level factors to be extended, which are commonly utilized in response surface modeling and optimization. The most significant benefit of this kind of optimization model is its accuracy, as the three-level factorial experiment is not required to construct a second-order quadratic model^34,35^. The effects of various factors such as methanol to the oleic acid molar ratio (MOMR), catalyst weight percentage, temperature, and time were investigated systematically.

**Figure 1:**
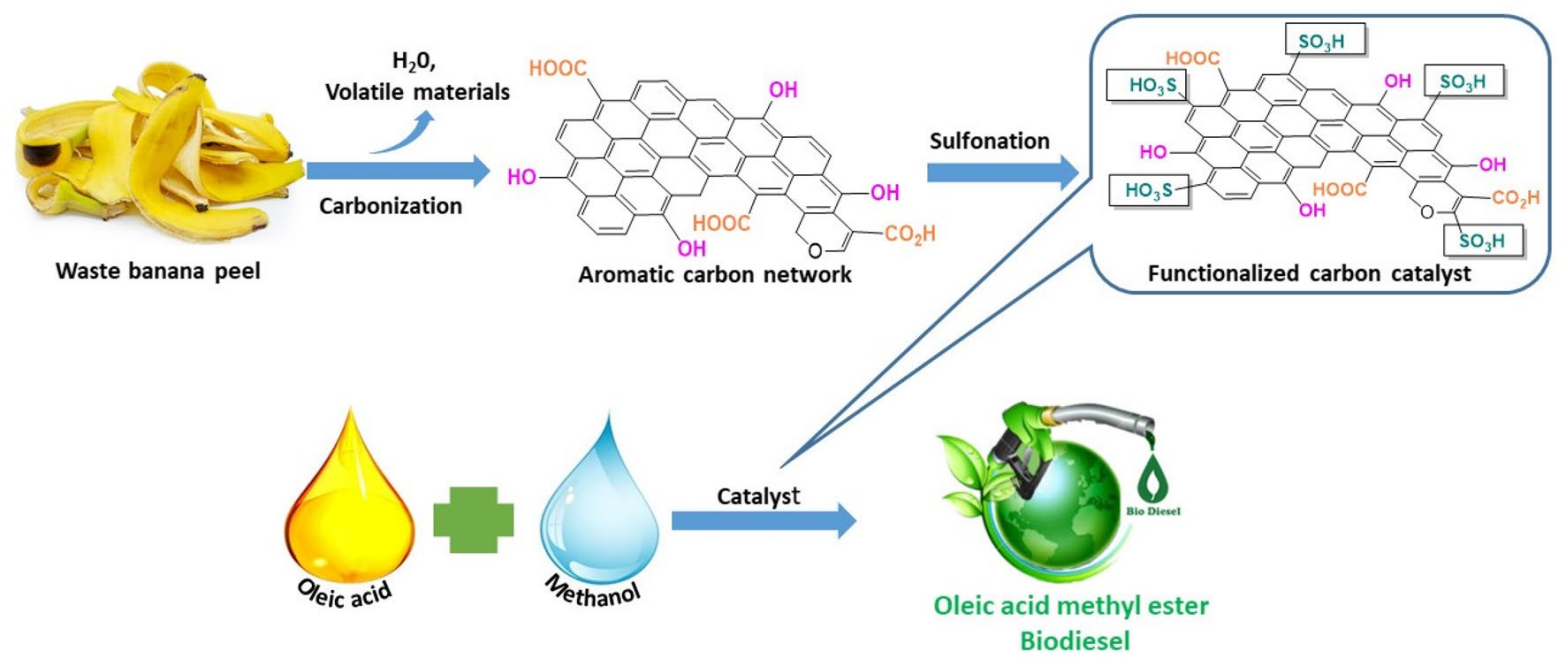
Production of biomass based catalyst for catalystic biodiesel production.

According to the report of Statista 2021, The largest fruit market in the entire globe is the banana market. The production of bananas was 124.98 million metric tonnes in 2021^36^. In addition, as a waste, it is quite expensive to dispose of away banana peel. Hence its beneficial usage would boost the economy of bananas in general^37^. Although a carbon-based solid catalyst made from waste biomass has the potential to solve many issues related to biodiesel production, its use on an industrial scale is constrained by the expense associated with catalyst fabrication^38^. For the industrial production of biodiesel, it is therefore highly desired to use a straightforward, economic, and ecologically friendly strategy that uses a waste biogenic heterogeneous catalyst^39^. Carbon materials such as banana peels are generally inexpensive and easily available, and they can be readily functionalized with –SO_3_H groups by treating them with concentrated H_2_SO_4_, resulting in sulfonated carbon catalysts. The acid-catalyzed esterification of oleic acid, normally present in natural oil, has become a paradigm in biodiesel research. Thus, the current study examines a novel method of using sulfonic acid functionalized banana peel (denoted as BP-SO_3_H-10-16-80 as a heterogeneous catalyst, as shown in Fig. 1), which can be regarded as more economical and effective, to convert oleic acid to biodiesel.

## Experimental methodology

### Materials and methods

Banana peels *(Musa acuminata*) were collected from Kolasib, Mizoram (24° 13′ 36.3792″ N, 92° 40′ 39.9″ E), India. The required permissions were obtained for collecting banana peels, and this study complies with relevant institutional, national, and international guidelines and legislation. The collected banana peels were dried thoroughly in the sunlight. Oleic acid (Analytical Reagent Grade, purity ≥ 99%) was purchased from Sigma Aldrich. Methanol (99.8%), H_2_SO_4_ (98.07%), BaCl_2_ (99.95%), NaHCO_3_ (≥ 98.7%) and were purchased from Merck. Double distilled water was used throughout the experiments. All the solvents and chemicals used were of analytical grade, bought from commercial sources, and utilized without purification.

### Preparation of BP-SO_3_H catalyst

Four batches of banana peel-supported sulfonic acid (BP-SO_3_H) catalysts were prepared by varying the banana peel-to-sulfuric acid ratio, reaction time, and temperature. Catalysts prepared in the initial batch include dried banana peel powder (1 g) mixed thoroughly with conc. H_2_SO_4._ Banana peel powder: H_2_SO_4_ ratios (g L^−1^) of 1:5, 1:10, 1:15, and 1:20 were used, whereas the reaction temperature varied between 80 and 120 °C while reaction time was monitored for 16, 18, 20, 22, and 24 h. In order to remove residual sulfate ions in the filtrate, 50–60 mL of deionized water was added to the mixture. It was washed several times with hot deionized water until no residual sulfate ions could be detected (a 6 mol L^−1^ solution of BaCl_2_ was used for testing). The resultant sulfonic acid functionalized banana peel (BP-SO_3_H) was dried in an oven overnight. All the synthesized BP-SO_3_H catalysts were assigned a code based on their synthesis procedure; BP-SO_3_H-X-Y-Z, where X is the wt/volume ratio of banana peel/H_2_SO_4_, Y is the 'in 'situ' hydrothermal-sulfonation time, and Z stands for reaction temperature. Accordingly, the catalyst prepared using a 1:10 (wt/volume) banana peel/sulfuric acid ratio, hydrothermal sulfonation time of 16 h, and reaction temperature of 80 °C were designated as BP-SO_3_H-10-16-80.

### Catalyst characterization

Based on the modified Boehm titration procedure, the density of –COOH, –SO_3_H, and –OH groups on the surface of BP-SO_3_H was determined^40,41^. The total acid density was determined using basic solutions of NaOH. The acid density of –COOH and –SO_3_H were calculated using NaHCO_3_ and NaCl, respectively. Equation (1) was used to calculate the total surface acid density of the catalyst.

1 $$n=\frac{{n}_{\text{HCl} }}{{n}_{B}} \left[B\right]{V}_{B}-(\left[\text{HCl}\right]{V}_{\text{HCl}}-[\text{NaOH}]{V}_{\text{NaOH}}) \frac{{V}_{B}}{{V}_{aliquot}}$$

where, $\frac{{n}_{\text{HCl} }}{{n}_{B}}$ is the molar ratio of HCl to base reacted; $\left[B\right]$ and ${V}_{B}$ are the concentration and volume of the reaction base mixed with the catalyst, respectively; V_aliquot_ is the volume of the aliquot taken from the ${V}_{B}$, [HCl] and ${V}_\text{HCl}$, are the concentration and volume of acid used in the acidification, and [NaOH] and ${V}_\text{NaOH}$ are the concentration and volume of the NaOH used in the titration.

A 40 kV generator voltage and a tube current of 100 mA were used to obtain powder X-ray diffraction (XRD) patterns at 2θ = 10–60° on an X'Pert Pro diffractometer (PAN analytical, Netherland). Before degassing for 10 h at 150 °C, the Brunauer–Emmett–Teller (BET) analysis was performed on a surface area and porosity analyzer (Micromeritics ASAP 2010, USA). A JEOL JSM-7600F (Japan) microscope was utilized for energy-dispersive X-ray spectroscopy (EDX) and scanning electron microscopy (SEM) at 1500× magnification power, 80 mA beam current, and 20 kV. The catalyst was disseminated in ethanol and dropped onto a Cu grid drop-wise before being dried in an oven. An electron microscope (JEM-2100, 200 kV) from JEOL (Tokyo, Japan) was used to record high-resolution transmission electron microscopy (HRTEM). Thermo gravimetric analysis (TGA) was performed in the temperature range of 50–600 °C under the continuous flow on N_2_ using a Perkin-Elmer instrument with model no TGA 4000 (Shelton, USA). A Nicolet 6700 spectrophotometer (Nicolet Instrument Corporation, USA) was used to record Fourier transform infrared spectra (FTIR) on the KBr pellet. Thermo Fisher 'Scientific's ESCALAB Xi^+^ (USA) device with Al Kα radiation was used for the X-ray photoelectron spectroscopy (XPS) analysis.

### Esterification of oleic acid

**Figure 2:**
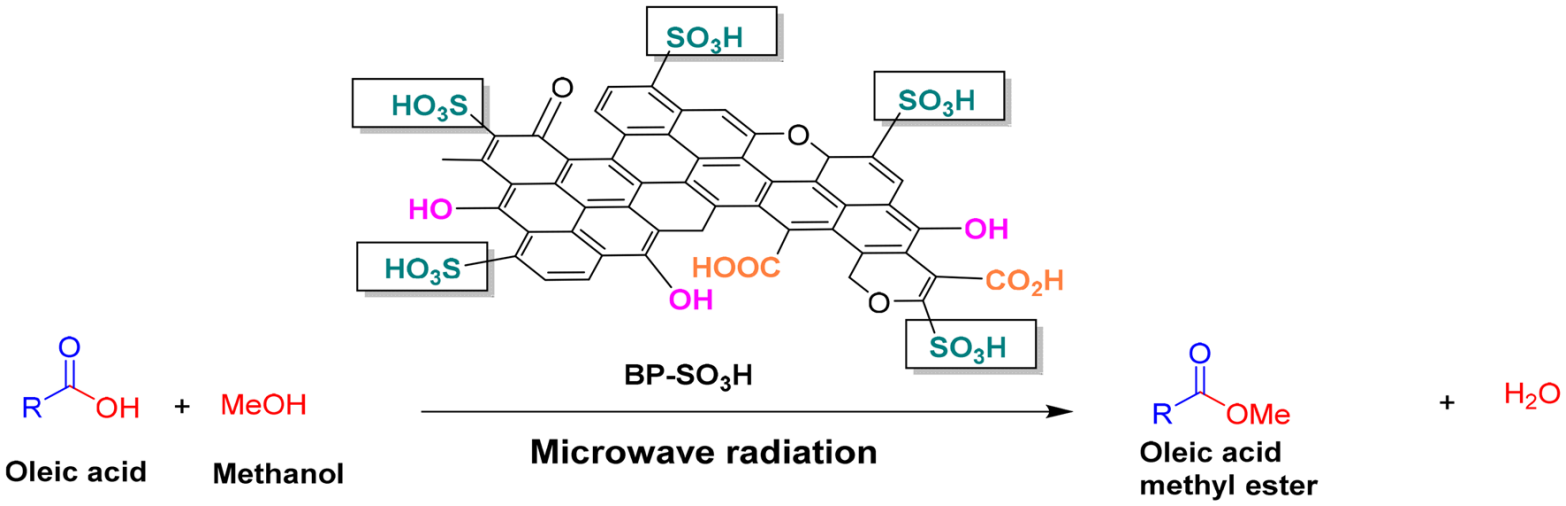
Oleic acid esterification using BP-SO_3_H as a heterogeneous catalyst.

The esterification reaction was carried out under microwave irradiation under different conditions (50–90 °C, 25–65 min, 2–10 catalyst wt% (with respect to OA), 5:1–25:1 MOMR; Fig. 2). Optimized conditions (see “Results and Discussion”) were methanol (20 mmol) and OA (1 mmol) in a 20:1 molar ratio and 8 wt% catalyst (0.0225 g) at 80 °C for 55 min. Thin-layer chromatography (TLC) was used to monitor the progress of the reaction and the formation of fatty acid methyl ester (FAME). The newly synthesized biodiesel was filtered when TLC showed complete conversion. The excess amount of methanol in the compound was evaporated using a rotary evaporator followed by a suction pump. ^1^HNMR analysis (Fig. S4) and Gas chromatography (GC), Fig. S5, were used to confirm the formation and purity of the biodiesel or fatty acid methyl ester product. The biodiesel quality (Table S1) was studied using the conventional American Society for Testing Materials (ASTM).

### Parametric optimization by response surface methodology (RSM)

Central composite design (CCD) efficiently fits experimental data using a second-order model. In this manner, variables are structured at three levels, each uniformly spaced, i.e., Low, intermediate, and high values represented by − 1, 0, and + 1, respectively^42^. It is more successfully utilized to construct a model that can be tested for accuracy using statistical analysis of variance (ANOVA) is a software-guided technique. This work uses CCD to measure model accuracy and how interacting factors affect the results^43^. The MeOH: OA molar ratio (A), catalyst loading (B), time (C), and temperature (D) were all investigated in the biodiesel manufacturing process. In total, 30 experiments were designed. To determine the performance of the components and their interaction affects the efficiency (optimal response) of biodiesel production, a quadratic polynomial equation (Eq. 2) is applied.

2 $$\begin{aligned} {\text{Biodiesel Yield }}\left( {\text{Y}} \right) & = \alpha_{{\text{o}}} + \, \alpha_{{1}} {\text{A }} + \, \alpha_{{2}} {\text{B }} + \, \alpha_{{3}} {\text{C }} + \, \alpha_{{4}} {\text{D }} \hfill \\ & \quad + \alpha_{{{12}}} {\text{AB }} + \, \alpha_{{{13}}} {\text{AC }} + \, \alpha_{{{14}}} {\text{AD }} + \, \alpha_{{{23}}} {\text{BC }} + \, \alpha_{{{24}}} {\text{BD }} \hfill \\ & \quad + \alpha_{{{34}}} {\text{CD }} + \, \alpha_{{{11}}} {\text{A}}^{{2}} +_{{}} \alpha_{{{22}}} {\text{B}}^{{2}} + \, \alpha_{{{33}}} {\text{C}}^{{2}} + \, \alpha_{{{44}}} {\text{D}}^{{2}} \hfill \\ \end{aligned}$$

where α_o_ is the intercept term, α_1–4_ are the coefficients of the linear terms, α_12-14_, α_23_, α_24,_ and α_34_ are coefficients of the interaction terms, and α_11_, α_22_, α_33,_ and α_44_ are coefficients of the quadratic terms. A–D are coded factors.

### Biodiesel product analysis

The biodiesel or fatty acid methyl ester was purified using column chromatography and then analyzed by ^1^H NMR and ^13^C NMR for product confirmation and purity. On a Bruker Avance II 700 MHz spectrometer (Fällanden, Switzerland), the ^1^H NMR and ^13^C NMR spectra of the produced biodiesel were recorded using tetramethylsilane (TMS) as an internal reference. Using the integrals for methoxy and methylene groups (${\mathrm{A}}_{\mathrm{Me}}$ and ${\mathrm{A}}_{{\mathrm{CH}}_{2}}$, respectively in Eq. (3)), biodiesel conversion was determined by the equation derived by Knothe and Kenar.

3 $$Conversion\,\left(\%\right)= \frac{{2A}_{Me}}{{3A}_{{CH}_{2}}}\times 100$$

where *C* indicates the triglyceride to biodiesel (fatty acid methyl ester) conversion percentage (%), *A*_Me_ denotes the integration value of the methyl esters, and ${A}_{{CH}_{2}}$  is the integration value of the methylene protons. Factors 2 and 3 ascribe to the proton number in methylene and the proton number in the methyl ester, respectively. The biodiesel yield was calculated by the equation given by Leung and Guo as given in Eq. (4).

4 $$Yield (\%)= \frac{Weight \; of \; methyl \; oleate \; produced}{Weight \; of \;oleic \; acid \; used} \times 100$$

### Kinetic study of the reaction

Considering the reaction to be a Pseudo-Homogeneous (P–H) model, the rate of the reaction can be expressed as

5 $$-\left(\frac{{dC}_{A}}{dt}\right)={k C}_{A}^{a}\,{C}_{B}^{b}- {k}^{^{\prime}}{C}_{E}^{e}\,{C}_{W}^{w}$$

where $k$ and ${k}^{^{\prime}}$ are the reaction rate constants, C_A_, C_B_, C_E_ and C_w_ are the concentration of oleic acid, methanol, methyl ester and water respectively; a, b, e, and w are the respective reaction orders. Since the concentration of methanol is too high with respect to other concentration terms, thus ${C}_{B}^{b}$ is considered to be constant. Also, $k$ is larger than ${k}^{^{\prime}}$^44,45^, so Eq. (5) can be reduced to (Eq. 6).

6 $$-\left(\frac{{dC}_{A}}{dt}\right)= {kC}_{A}^{n}$$

7 $${C}_{A}= {C}_{{A}_{o}}(1-X)$$

${C}_{{A}_{o}}$ and $X$ refers to initial concentration of oleic acid and fractional biodiesel yield, respectively. Furthermore, Eq. (7) can be written as Eq. (8).

8 $$-\left(\frac{dx}{dt}\right)= \left(\frac{k}{{C}_{{A}_{o}}}\right) {\left[{C}_{{A}_{o}}\left(1-X\right)\right]}^{n}= {k}_{1} {\left[{C}_{{A}_{o}}\left(1-X\right)\right]}^{n}$$

9 $$\mathrm{ln}\left(1-X\right)= -kt$$

10 $$\frac{X}{\left(1-X\right)}=k{C}_{{A}_{o}}t$$

where, $\left(\frac{k}{{C}_{{A}_{o}}}\right)= {k}_{1}$ and for n = 1, the above equation, Eq. (8) can be integrated as Eq. (9). Similarly, for n = 2, second order reaction, Eq. (8) can be integrated to a simplified equation, Eq. (10). As given in Eq. (11), the activation energy (Ea) of the reaction was determined using the Arrhenius equation in which the rate constants at different temperatures were used (50–90 °C)^46^.

11 $$\mathrm{ln k}= -\frac{Ea}{RT}+\mathrm{ln}A$$

Here, the reaction temperature is denoted by T, the pre-exponential factor by A and R is 8.314 × 10^–3^ kJ K^−1^ mol^−1^.

### Test for heterogeneity and reusability of the catalyst

The hot filtration method (Sheldon's test)^47^ was employed for the heterogeneity test of the BP-SO_3_H-15-18-100 catalyst. After 35 min, filtration separated the catalyst from the reaction mixture. Then, the reaction was continued for another 30 min with a catalyst-free and filtered reaction mixture; further TLC and GC were employed to monitor the progress of the reaction.

TLC conditions: TLC plates were made from microscope slides of 76.2 × 25.5 mm and a thickness of 1.2 mm. Silica was used as the adsorbent (stationary phase), and 20% ethyl acetate solution (mobile phase) was used to run the TLC plates.

GC conditions: For GC analysis, the sample was diluted five times with ethanol. Conditions for a GC include an HP-5MS quartz capillary (30 mm × 0.25 0.25 mm × 0.25 m), a column temperature range of 70–260 °C, a programmed temperature rate of 5 °C/min, a column flow rate of 1.0 mL/min, an inlet temperature of 260 °C, a pressure in front of the column of 100 kPa, an injection volume of 0.40 L, transmission line temperature of 250 °C, an ion source temperature of 220 °C, a quadrupole temperature of 140 °C, and a mass scanning range of 35–500 amu.

Reusing a solid heterogeneous catalyst is one of its most important characteristics. Oleic acid esterification using recycled catalyst was carried out under optimized conditions to test the catalyst's reusability. The recovered catalyst was filtered and washed with methanol after each catalytic run. It was then dried overnight at 80 °C in a vacuum oven before being utilized in the next cycle.

## Results and discussion

### Total acidity test using Boehm titration

The catalyst of 100 mg was dissolved in 40 mL of 0.05 M NaOH and stirred for 24 h. The mixture was then filtered, and 10.00 mL of aliquot was taken and acidified with 20.00 mL of standardized 0.05 M HCl solution. Further, phenolphthalein indicator was added and titrated with standardized 0.05 M NaOH. The same procedure was repeated to get the concordant value. The above procedure was followed to determine the acid density of –COOH using NaHCO_3_ as a basic solution and the acid density of SO_3_H 2 M NaCl solution. Finally, the acid density of –OH was calculated by subtracting the acid density of –COOH and –SO_3_H from the total acid density.

### Catalyst composition in relation to sulfonation parameters

A total of four batches of BP-SO_3_H catalysts with banana peel and conc. H_2_SO_4_ in the ratio of 1:5, 1:10, 1:15, and 1:20 (wt in g/volume in mL) were prepared with different reaction times ranging from 16 to 24 h by varying temperatures from 80 to 120 °C in a Pyrex bottle to determine the level of sulfonation. In the sulfonation process, the ratios of sulfonating agent to biomass precursor ratio are known to impact the total acid density of the catalyst^48^. As the sulfuric acid added in the first batch (1:5) was much less, the S content and overall acidity were quite low compared to the later batches. The overall acid density in 1:10, 1:15, and 1:20 were studied separately, and it was found that 1:15 (wt in g/volume in mL) is the optimum condition, while continued addition of sulfuric acid resulted in a drop in both parameters. This can be attributed to several factors. Dehydration, which occurs at high sulfuric acid concentrations, will remove –OH and –COOH groups, thus reducing total acid density^49^. With the increase in dehydration, aromatization and cyclization proceed more quickly, decreasing the amount of surface that may be available for sulfonation, ultimately reducing S content. The ratio of 1:15 had the highest acid density (4.3 mmol g^−1^) and sulfur content (4.62 wt%). Hence it was concluded that when sulfuric acid is loaded at a higher concentration during sulfonation, more -SO_3_H groups can be attached to the catalyst surface.

Increasing the time of sulfonation resulted in an increase in the acid densities of the catalysts until an optimal time of 18 h was reached. However, a further increase in the sulfonation time beyond the optimal point did not influence the overall acid density values of the catalyst^50,51^.

With the increase in the sulfonation temperature, the –SO_3_H and phenolic –OH density of the catalyst increases significantly up to a certain temperature. However, BP-SO_3_H-15-18-120 had lower amounts of –SO_3_H and phenolic –OH than BP-SO_3_H-15-18-100 because high sulfonation temperature resulted in a higher degree of acid dehydration, which led to reduced C–H and C–OH bonds that are thereby producing harder carbon materials which provide lesser edges for sulfonation^50,52^. The preparation and activities of different catalysts used for biodiesel synthesis are presented in Table 1, whereas the complete experimental biodiesel yields using the optimized catalyst (BP-SO_3_H-15-18-100) are shown in Table 2.

**Table 1:** Reactivity of different catalysts in oleic acid esterification under microwave irradiation.

Sl. no	Catalyst	Sulfur content^a^	Total acidity (mmol g^−1^)^b^	Yield (%)
1	BP-SO_3_H-15-18-80	1.12	1.53	45.5
2	BP-SO_3_H-15-18-100	4.62	4.30	97.9
3	BP-SO_3_H-15-18-120	1.35	1.72	50.1
4	BP-SO_3_H-15-16-100	4.10	3.39	90.5
5	BP-SO_3_H-15-20-100	3.99	3.16	89.3
6	BP-SO_3_H-10-18-100	3.81	3.07	77.2
7	BP-SO_3_H-10-16-120	3.73	2.78	75.6
8	BP-SO_3_H-5-18-100	1.92	2.21	60.5
9	BP-SO_3_H-10-20-100	3.68	2.85	72.7
10	BP-SO_3_H-20-18-100	4.01	3.25	83.4

Under the reaction conditions of: MOMR 20:1, catalyst loading of 8 wt% (w.r.t oleic acid), the temperature of 80 °C, and reaction time of 55 min.

^a^Based on SEM–EDX analysis.

^b^Analysis using Boehm titration.

**Table 2:** The design matrix includes experimental variables (A–D) and predicted and actual biodiesel yield.

Run no	MOMR (A)	Catalyst loading (wt%) (B)	Time (min) (C)^a^	Temperature (°C) (D)	Predicted biodiesel yield (%)	Actual biodiesel yield (%)
1	15	10	45	90	77.66	76.9
2	15	6	45	70	61.23	61.4
3	15	10	65	90	76.97	76.8
4	20	8	35	80	79.28	79.7
5	15	6	65	90	81.68	80.9
6	20	12	55	80	85.18	85.3
7	15	10	45	70	80.27	79.8
8	25	6	45	70	76.99	76.5
9	25	10	65	90	76.08	75.7
10	25	10	65	70	78.84	77.9
11	20	8	55	60	75.30	75.1
12	20	8	55	80	97.45	97.9
13	20	8	55	80	97.45	97.0
14	10	8	55	80	65.03	65.4
15	20	8	55	80	97.45	97.9
16	25	10	45	70	82.73	83.3
17	20	4	55	80	84.15	84.9
18	20	8	55	100	81.83	82.9
19	20	8	55	80	97.45	97.1
20	15	10	65	70	83.18	84.1
21	20	8	55	80	97.45	97.0
22	15	6	45	90	70.52	70.8
23	25	6	65	70	84.95	85.5
24	30	8	55	80	79.90	80.4
25	25	10	45	90	83.57	83.7
26	15	6	65	70	75.99	75.2
27	25	6	45	90	89.73	88.6
28	20	8	75	80	86.55	87.0
29	25	6	65	90	94.09	93.9
30	20	8	55	80	97.45	97.8

^a^Reaction time in mins under microwave-irradiation.

### Characterization of catalyst

Powder X-ray diffraction analysis (PXRD) was used to evaluate the crystallinity of the biomass-derived BP-SO_3_H catalyst, and the results were analysed. According to Fig. S1a given in supplementary information (SI), there is no particular diffraction peak in the whole pattern. A broad peak is obtained around 2θ = 23°, suggesting that amorphous catalysts were produced during a green synthesis process^53^. It is generally accepted that sulfonated catalysts have amorphous structures consisting mostly of sheets of polycyclic aromatic carbons coated with –SO_3_H, –OH, and –COOH^54^.

TGA of BP and BP-SO_3_H (Fig. S1b) displays significant changes under the thermal responses at a temperature ranging from 30 to 800 °C. In the TGA of BP, a small mass loss from the temperature range 30–100 °C could be ascribed to the loss of moisture, while a major mass loss from 140 to 430 °C could be attributed to the evolution of volatile compounds present in the BP. However, in the TGA of BP-SO_3_H, mass loss are accounted for the loss of adsorbed water from temperature range of 50–100 °C followed by mass loss from range of 250–500 °C due to the decomposition of –COOH, –SO_3_H, and –OH groups^55^.

As shown in Fig. S1c, the N_2_ adsorption–desorption isotherm of BP-SO_3_H catalyst exhibited a type IV isotherm with H_3_-type hysteresis loop, suggesting the presence of non-homogeneous mesoporous material in the catalyst. Pore size distribution analysis by BJH (Barrett, Joyner, and Halenda) showed a distribution of 2.205 nm. The pore volume and surface area of catalyst BP-SO_3_H were both within the ranges predicted for this type of material, at 0.016 cc g^−1^ and 14.024 m^2^ g^−1^, respectively^56^.

In order to detect the functional groups present, FTIR analysis of BP-SO_3_H-15-18-100 along with BP, recovered BP-SO_3_H was performed (Fig. S1d). The analysis showed that a 1028 cm^−1^ peak is associated with SO_3_^−^ symmetric stretching^57^ found in both the sulfonated and recovered catalyst, indicating the presence of the sulfonic group even after 5-times reuse of catalyst. Peaks at 1700 cm^−1^ and 1613 cm^−1^ were attributable to carbonyl stretching and C=C stretching in aromatic rings, respectively^58^. The peak at 2921 cm^−1^ and 3300 cm^−1^ corresponds to C–H and O–H stretching vibrations, respectively^59^.

In the SEM and TEM images of raw BP Fig. (S2a,be,f), large particles of irregularly compacted layers could be seen. On the surface of the clay particles, there is a wide distribution of dispersed spherical grains, which may be associated with the presence of organic substances. However, in contrast, the surface of the clay particles of BP-SO_3_H (Fig. S3c,d) appears to be smoother than the particles of BP. This result also corroborated with the TEM images (Fig. S2g,h) of BP-SO_3_H which shows agglomeration of dense particles leading to a spherical structure. This possibly could be due to sulfonation using H_2_SO_4_ which might be the effect of the acid in oxidizing organic matters and dissolving impurities^32^. The EDX analysis results also support the observation, as the S content was found to be 4.62 wt% (1.4437 mmol g^−1^) (Fig. S3g). Before sulfonation (Fig. S3d,e), there was a quantitative presence of C, O, Mg, Si, P, Cl and K, while after sulfonation in the SEM–EDX spectrum of BP-SO_3_H (Fig, S3f,g) it could clearly be seen that there is increase of carbon amount with less irregularity and increase of porosity due to simultaneous carbonization and sulfonation. The elemental mapping also confirmed the distribution of carbon (red), oxygen (yellow), and sulfur (green) for the BP-SO_3_H catalyst.

**Figure 3:**
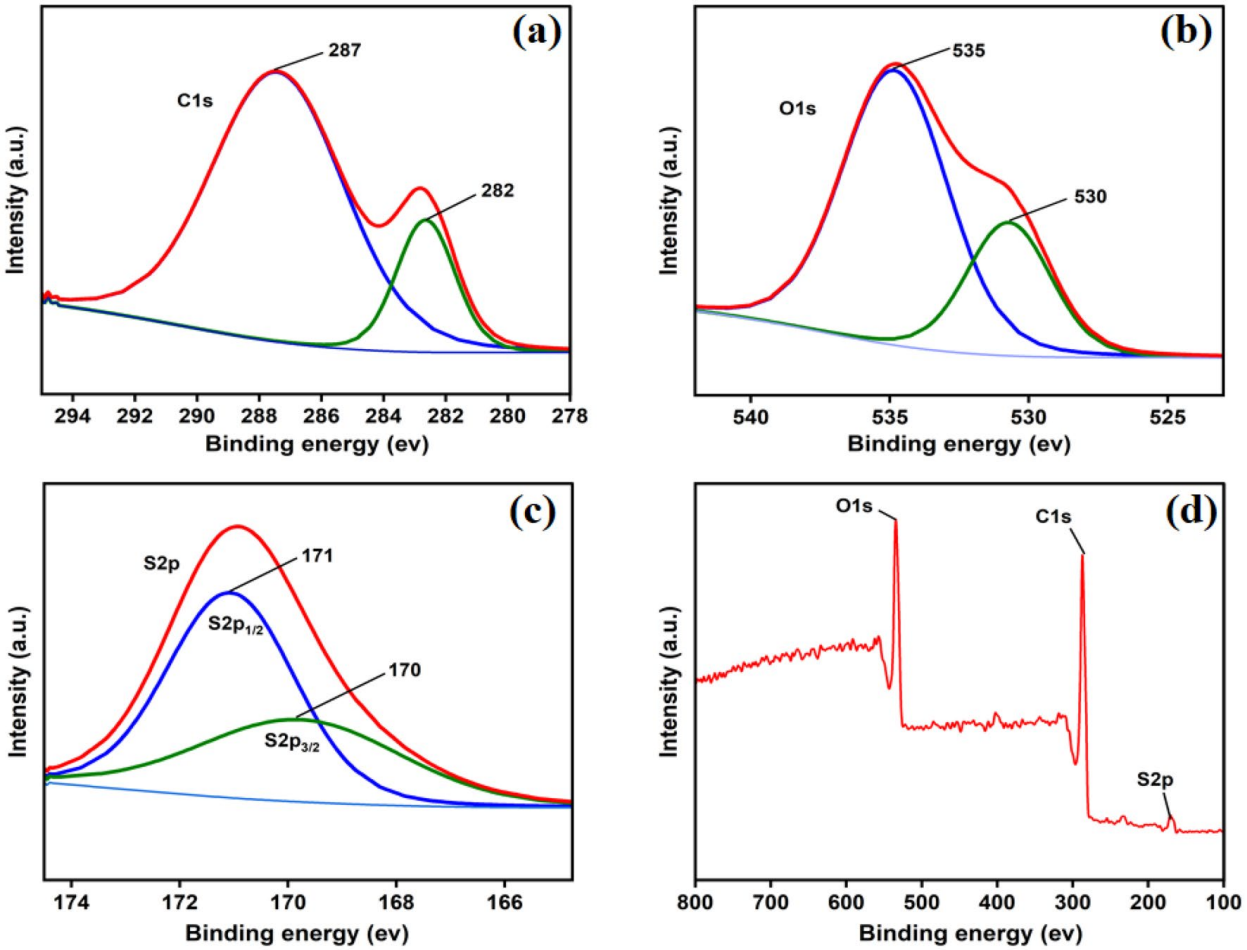
XPS analysis of BP-SO_3_H catalyst. Experimental spectra with deconvolution for C1s (**a**), O1s (**b**), S_2_p (**c**) regions and XPS survey spectrum of fresh BP-SO_3_H (**d**).

Figure 3 shows XPS analysis which was carried out on the acid-functionalized catalyst BP-SO_3_H. C, O, and S were visible in the spectrum. In the deconvoluted spectrum of the C1s region, major peaks at 287 eV and 282 eV are assigned to C=O and C=C, respectively (Fig. 3a). Similarly, the O1s (Fig. 3b) region showed peaks at 535 and 530 eV for C=O and C–O, respectively^56^. XPS also demonstrates the presence of a sulfonic group in the catalyst. As seen in Fig. 3c, the strong S2p peak at 170 eV corresponds to the –SO_3_H group^60^. This means that most of the S in the sample is in the form of –SO_3_H. On the other hand, a peak at 171 eV (weak) exists, which suggests a minute quantity of –O–SO_3_H^61^.

### Data analysis and modeling using the response surface method (RSM)

The relationship between responses (% yield) and reaction variables A-D (see "Parametric optimization by response surface methodology (RSM)" section) was analyzed using RSM. CCD technique is a partial factorial design method with the center point amplified with a gathering of the axial point that grants evaluation of non-linearity in the predicted model. For the developments of CCD, the number of experiment (N) is calculated by N = 2^n^ + 2n + m, where n is the number of independent variables and m is the number of replicated central point^42^. For the current system, n = 4 and m = 6. This forms a set of 16 cube points, 8 axial points, and six repeated center data; thus total 30 experiments run (refer to Table 2), which are analyzed in a randomized order where axial point, α = 0.05. A series of thirty experiments were conducted and the results of each CCD experiment along with biodiesel yield are shown in Table 2.

The actual biodiesel yield in experiments ranged from 61.4 to 97.9%. Moreover, Eq. (8) describes the biodiesel yield affected by the coded factors A–D (see the experimental variables A–D in Table 3).

12 $$\begin{aligned} {\text{Biodiesel yield }}\% \, \left( {\text{Y}} \right)& = {97}.{45}\, + \,{3}.{\text{72A}}\, + \,0.{\text{2583B}}\, + \,{1}.{\text{82C}}\, + \,{1}.{\text{63D}} \hfill \\ & \quad - 3.{\text{32AB }} - {1}.{7}0{\text{AC}}\, + \,0.{\text{8625AD }} - {2}.{\text{96BC }} - {2}.{\text{97BD}} \hfill \\ & \quad - 0.{9}000{\text{CD }} - {6}.{\text{25A}}^{{2}} { - 3}.{2}0{\text{B}}^{{2}} - {3}.{\text{63C}}^{{2}} { - 4}.{\text{72D}}^{{2}} . \hfill \\ \end{aligned}$$

**Table 3:** Statistical results for the regression model of OA esterification.

Source of variance	Sum of squares	Df	Mean square	F-value	p-value	Remark	Accuracy test	
							Parameters	Value
Model	2703.73	14	193.12	295.47	< 0.0001	Significant	R^2^	0.9975
A	331.53	1	331.53	507.22	< 0.0001		Adjusted R^2^	0.9952
B	1.60	1	1.60	2.45	0.1383			
C	79.21	1	79.21	121.18	< 0.0001		Adequate precision	63.2437
D	64.03	1	64.03	97.96	< 0.0001			
AB	176.89	1	176.89	270.63	< 0.0001			
AC	46.24	1	46.24	70.75	< 0.0001			
AD	11.90	1	11.90	18.21	0.0007			
BC	140.42	1	140.42	214.84	< 0.0001			
BD	141.61	1	141.61	216.66	< 0.0001			
CD	12.96	1	12.96	19.83	0.0005			
A^2^	1070.00	1	1070.00	1637.06	< 0.0001			
B^2^	280.14	1	280.14	428.60	< 0.0001			
C^2^	362.09	1	362.09	553.98	< 0.0001			
D^2^	611.28	1	611.28	935.24	< 0.0001			
Residual	9.80	15	0.6536					
Lack of fit	8.75	10	0.8749	4.15	0.0650	Not significant		
Pure error	1.06	5	0.2110					
Cor total	2713.53	29						

### ANOVA study of the process

ANOVA was used to determine the statistical significance of the model equation and the effects of the terms and their interactions on biodiesel yield to evaluate the significance and fitness of the quadratic regression model. Table 3 indicates the ANOVA results for the yield of OA to biodiesel. At the 95% confidence level, the regression model's F-value (Fischer test, which determines the significance of the chosen model and individual parameter affecting the response) of 295.47 and p-value < 0.0001 (probability of error, with < 0.05 representing significance) shows that it is statistically significant^62,63^. As shown in Table 3, A, C, D, AB, AC, AD, BC, BD, CD, A^2^, B^2^, C^2^, and D^2^ are significant, while B is not a significant model term. Several statistical parameters were used to assess the accuracy of the regression equation, including the coefficient of determination (R^2^), the adjusted R^2^, and adequate precision.

As described by the regression model equation, the R^2^ value of 0.9964 indicates that the regression model can explain 99.7% of the variation in biodiesel yield. Thus, the developed model correlates well with actual and predicted biodiesel yields^64^. This was supported by the adjusted R^2^ of 0.9930, excluding non-significant terms in the model. The model yielded a precision of 63.35 (a value greater than 4 is always preferred)^65^. According to the value obtained, the signal is adequate, and the regression model is useful for directing the design space for the experimental results^64^. A value of ˂ 10% indicates that the experimental values correlate with the model's predicted values^66^.

**Figure 4:**
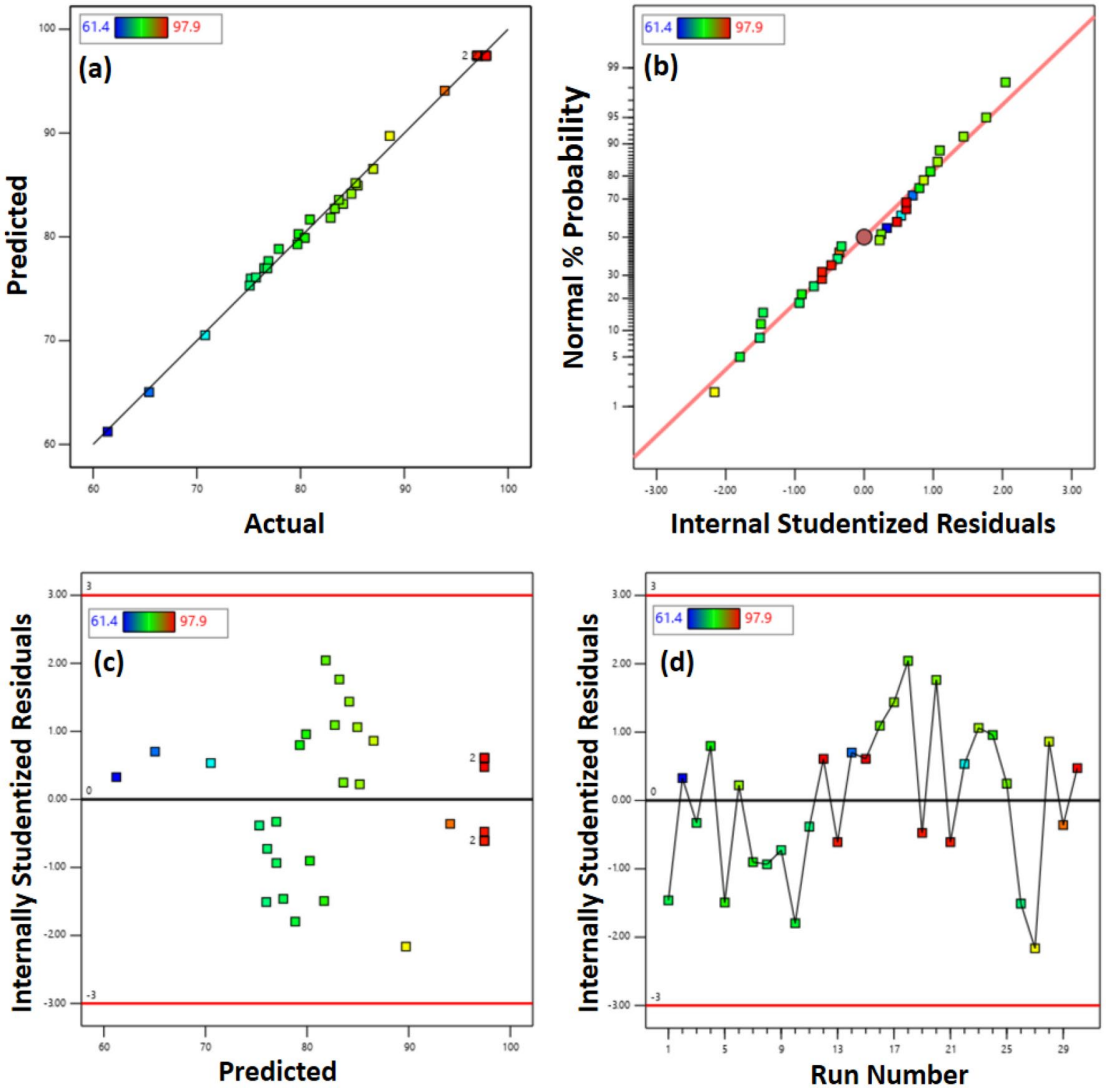
Diagnostic plots of (**a**) predicted % FAME yield versus actual % FAME yield, (**b**) Normal regression plot, (**c**) studentized residuals vs. predicted biodiesel yield, and (**d**) residual differences between predicted and actual yield of experimental runs.

Diagnostic plots (Fig. 4) were used to evaluate the quality of the regression model developed in this study. Figure 4a depicts a correspondence OA's actual and predicted yield. It indicates a good response estimation concerning changes in the independent variable A–D since data points are close to the fit regression line. It is crucial to check whether the regression model is acceptable by assessing if the residuals have a normal distribution. In experiments, residuals are the differences between actual and predicted values and represent how well the model fits the assumptions of ANOVA.

Figure 4b shows the plot of normal distribution versus studentized residuals. The plot implies that most of the data follow a straight line rather than the abnormal S-shape indicating studentized residuals follow a normal distribution^67^.

In Fig. 4c, the studentized residuals are displayed against the predicted yield. The residuals are distributed randomly in the plot, suggesting that the original observation is not related to the response values, as suggested by the model. As a result, the response parameter does not need to be transformed.

Figure 4d illustrates the residuals vs. the experimental run. Due to the presence of noise in the experiments, the residual differences between the experimental runs are large. The abscissa represents the number of experiments, whereas the ordinate represents the studentized residuals. All residuals fall within the range of 4.00, indicating that the fitted model accurately approximates all data with no errors recorded^68^.

**Figure 5:**
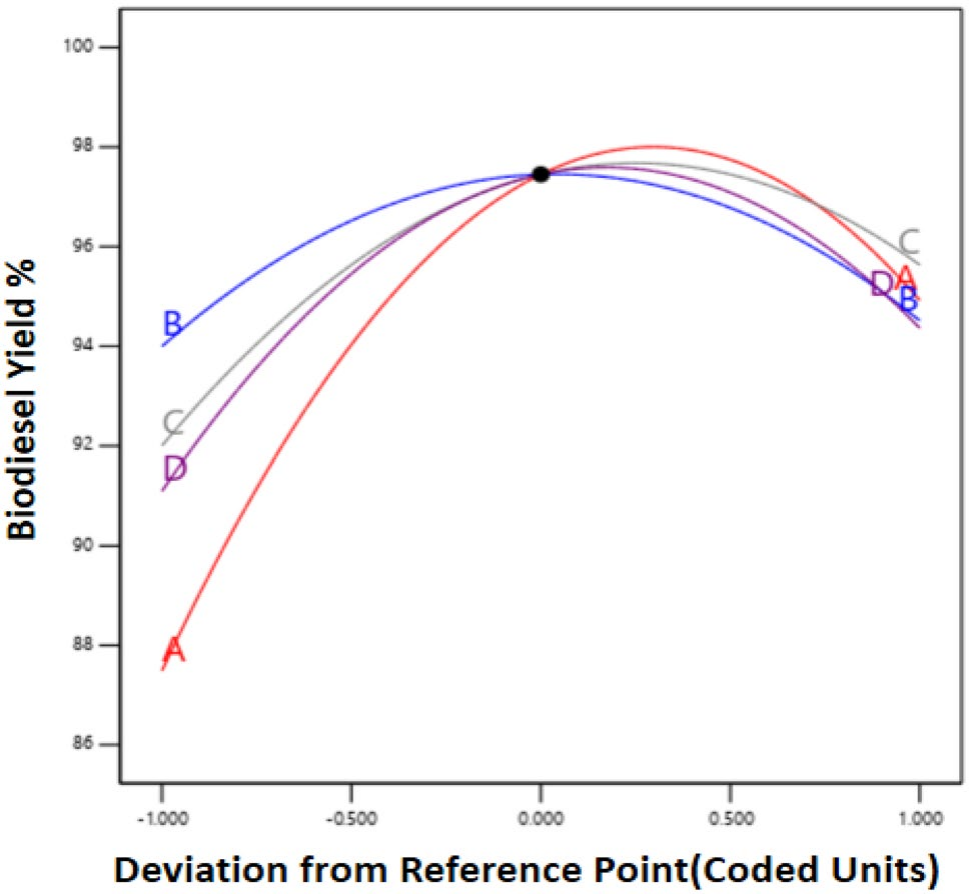
Perturbation plot exhibiting important esterification variables affecting OA biodiesel yield % using the microwave-assisted process.

Perturbation plots (Fig. 5) help classify the process variables' effect on yield while keeping the other process variables constant at an intermediate level^69^. The nature of the curvature represents the variability A–D factors on the biodiesel yield. The factor with a steeper slope more prominently affects biodiesel yield than with a flatter slope^70^. The plot (Fig. 5) illustrates factor A's prominent effect on biodiesel yield, followed by D, C, and B; similarities can be seen in ANOVA Table 3. Figure 5 shows that the molar ratio (A) at the lower level till the middle value is more sensitive; in contrast, the sensitivity is reduced between the middle and higher levels. Between intermediate to higher level parameter, B and D has a prominent effect on yield. Above the intermediate level, variation in biodiesel yield is moderate compared to the below-intermediate range. As per the ANOVA analysis, the molar ratio (A) is the most significant factor affecting the yield.

### Interaction effects of process input variables on OA biodiesel yield

**Figure 6:**
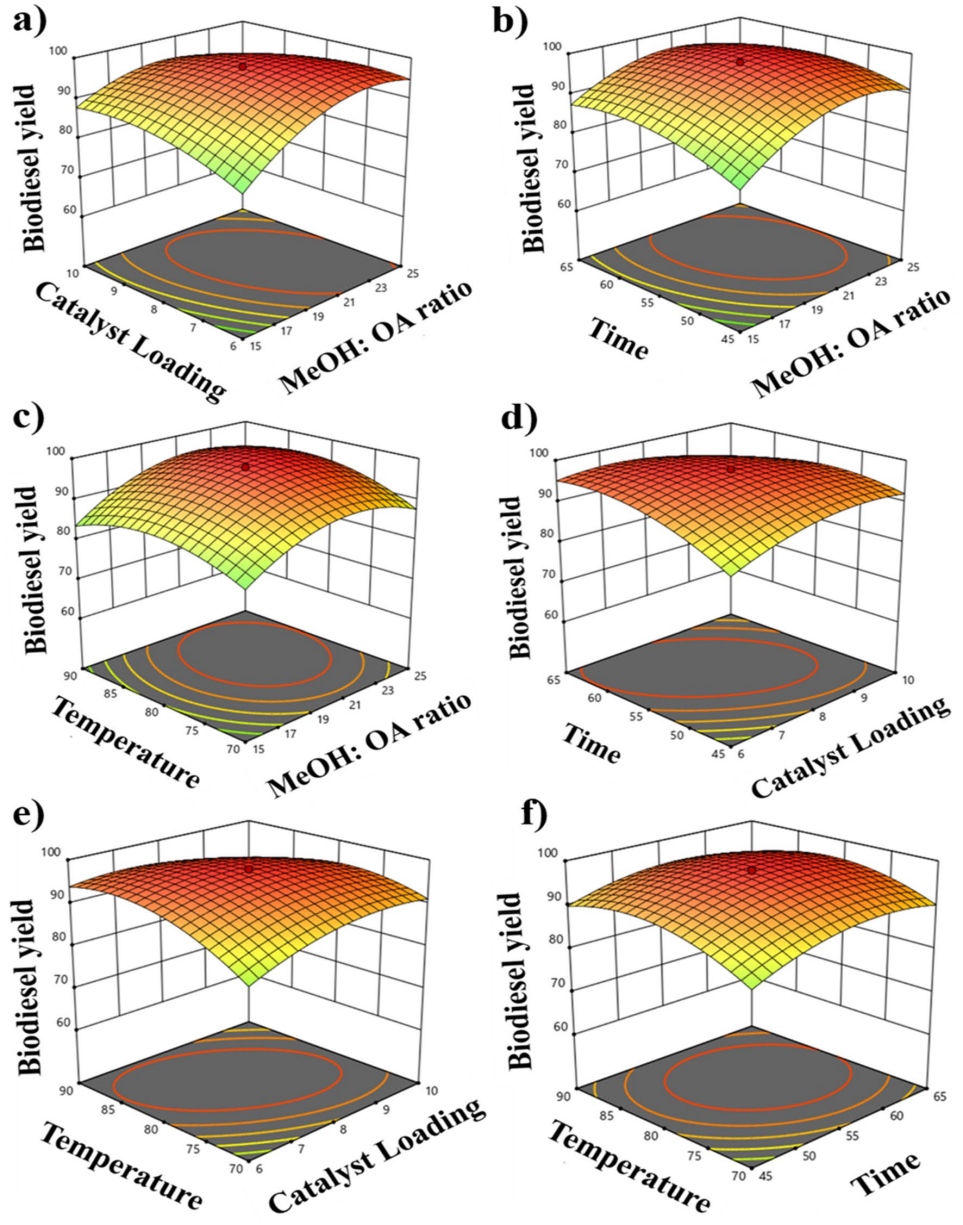
3D surface diagram for the interaction between the independent variables A-D and their effect on the efficiency of microwave-assisted biodiesel production from OA.

In order to measure the influence of independent parameters A–D (viz., A = MOMR; B = catalyst loading; C = time; and D = temperature) on experimental biodiesel yield obtained through microwave heating, 3D response surface plots were used^70^. The effect of MOMR and catalyst loading on biodiesel yield is shown in Fig. 6a. By increasing both the variables, biodiesel yield also increased until it reached its maximum of 8 wt% catalyst loading and a MOMR of 20:1. A moderate decrease in yield was observed when the reaction period was prolonged beyond optimum value due to esterification's reversibility^67^. According to the 3D response curve, the interaction between MOMR and catalyst loading significantly affects the reaction.

The relationship between MOMR and reaction time is depicted in Fig. 6b. By prolonging the reaction time from 45 to 65 min, no significant improvement was observed in the yield efficiency at low methanol to the oleic acid molar ratio (specify the range). However, it showed considerable variations, with a maximum yield at 20:1, whereas altering duration had little effect.

Figure 6c shows the effect of MOMR and temperature interaction on OA yield. The yield increases with increasing both factors until it reaches its center maximum of 98.2% at 20:1 MOMR and 80 °C, which declines modestly. This can be attributed to excessive methanol diluting the reaction mixture^71^ and thus allowing water to be introduced, reinitiating the reverse reaction^72^. MOMR and temperature interact strongly in this reaction, as seen in the 3D response curve.

Figure 6d illustrates the effect of the catalyst dosage and reaction time on biodiesel yield. With an increase in the amount of catalyst, oleic acid yield increased drastically as more active sites were provided. Despite the further increase in catalyst concentration, the maximum yield of oleic acid to methyl oleate was observed at 8 wt%. The maximum yield was observed at 55 min, after which it declined due to the reversibility of esterification reaction^73^.

Figure 6e shows the interaction of catalyst loading and temperature. The methyl oleate yield was highest at 8 wt% and 80 °C, respectively. Due to the endothermic nature of esterification, an increase in temperature resulted in initially increased yield^74^. At higher temperatures, there was a drop in yield due to methanol evaporation^75^.

Figure 6f illustrates the interaction of reaction time and temperature. As the temperature initially increased, methyl oleate yield increased to 98.2% at 80 °C and 55 min. Both factors show significant interaction. Considering these data, 55 min of reaction time, 80 °C of temperature, 8 wt% of catalyst loading, and a MOMR of 20:1 has been concluded as the optimal conditions for the given microwave-assisted esterification reaction.

### Analysis of ^1^H NMR and ^13^C NMR

Fig. S6a, given in the SI shows the ^1^H NMR spectrum of methyl oleate (biodiesel). A singlet signal at 3.62 ppm represents methoxy protons in the methyl ester. A triplet signal at 2.26 ppm represents the α-CH_2_ protons in the methyl ester (–CH_2_CH_2_COOMe). It is evident from these two peaks that there are methyl esters in biodiesel. A triplet signal of 0.84 ppm was observed as the result of the terminal methyl protons, a strong multiplet signal at 1.22 ppm was observed from the methylene protons of the carbon chain, a multiplet was observed at 1.57 ppm from the β-carbonyl methylene protons (-*CH*_*2*_CH_2_COOMe), a multiplet at 1.97 ppm and another at 5.30 ppm was detected from the olefinic hydrogen^76,77^. ^1^H-NMR revealed 99.5% biodiesel conversion by triplet integration at δ 2.28 ppm for methyl oleate (using the formula given in (Eq. 3))^78^. Furthermore, biodiesel yield of 97.9% was calculated using Eq. (4) under the same optimized condition.

Fig. S6b shows the ^13^C NMR spectrum of methyl oleate (biodiesel). The carbonyl carbon of the ester molecules (–*C*OOMe) is represented by the signal at 174.8 ppm, whereas the signal at 130.5 ppm represents the olefinic carbons. Methoxy carbons of methyl esters (–COO*Me*) are responsible for the signal at 52 ppm in the ^13^C NMR spectra of biodiesel. The fatty acid moiety's methylene and methyl carbons are found in the range of 14.6 to 34.6 ppm^79^.

### Kinetic study of oleic acid esterification

**Figure 7:**
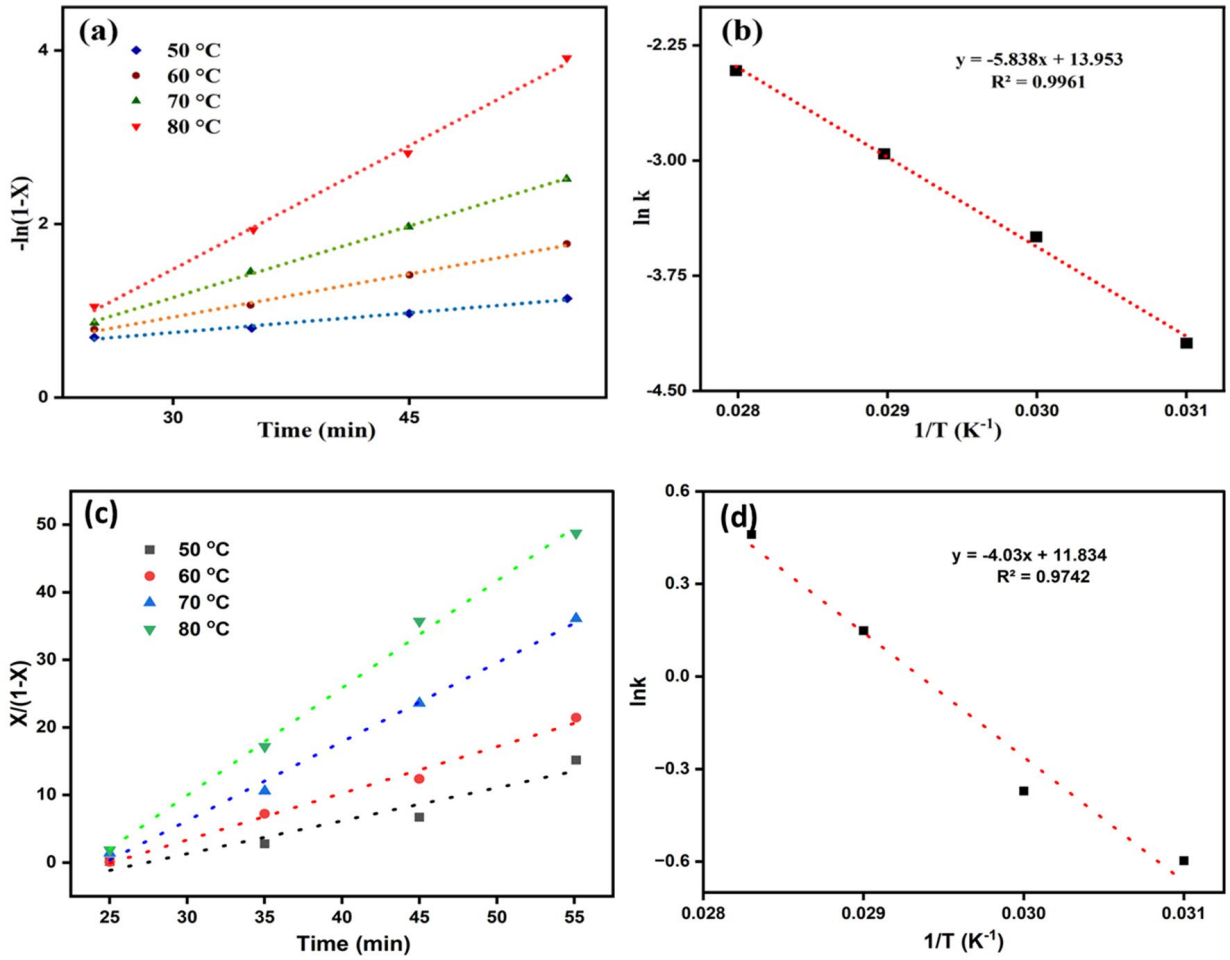
Plot showing the relationship between -ln(1-X) vs. time (**a**) and X/(1-X) vs. time (**c)**, where X is the biodiesel yield at various temperatures for the esterification reaction of oleic acid. (**b**) and (**d**) showing the corresponding Arrhenius plot of ln k vs 1/T.

Figure 7a,c showed a linear relationship between −ln(1−X) Vs. time and X/(1−X) Vs. time for reactions carried out at different temperatures ranging from 50 to 80 °C. The plots in Fig. 7a–d represent pseudo-first and second-order reaction kinetics based on the Eqs. (9) and (10), respectively. The activation energy was determined by different rate constants and the Arrhenius equation (Eq. 11). Upon comparing the linear regression coefficients (R^2^) it was found that the pseudo-first-order reaction kinetic model had a higher R^2^ value of 0.9961 than the second-order kinetic model having R^2^ value of 0.9742. This indicated that the constructed pseudo-first-order kinetic model is more plausible. The esterification reaction's activation energy (Ea) was determined by substituting the rate constants in the Arrhenius equation (Eq. 11). From the line's intercept and slope (−Ea/R), the pre-exponential factor and Ea for the reaction can be inferred, respectively^75^. From Fig. 7b, Ea was found to be 48.53 kJ mol^−1^, which comes in the range of 24.7–84.1 kJ mol^−1^^46,80^. It showed that the current catalyst induces a substantial decrease in activation energy compared to various other esterification catalysts. These include Al-Sr nanocatalyst (72.9 kJ mol^−1^)^81^, CaO/SiO_2_ (66.3 kJ mol^−1^)^80^ and H_3_PW_12_O_40_ (51.0 kJ mol^−1^)^82^. The pre-exponential factor in the present study was calculated to be 1.1 × 10^6^ min^−1^.

### Test for heterogeneity and catalyst reusability

**Figure 8:**
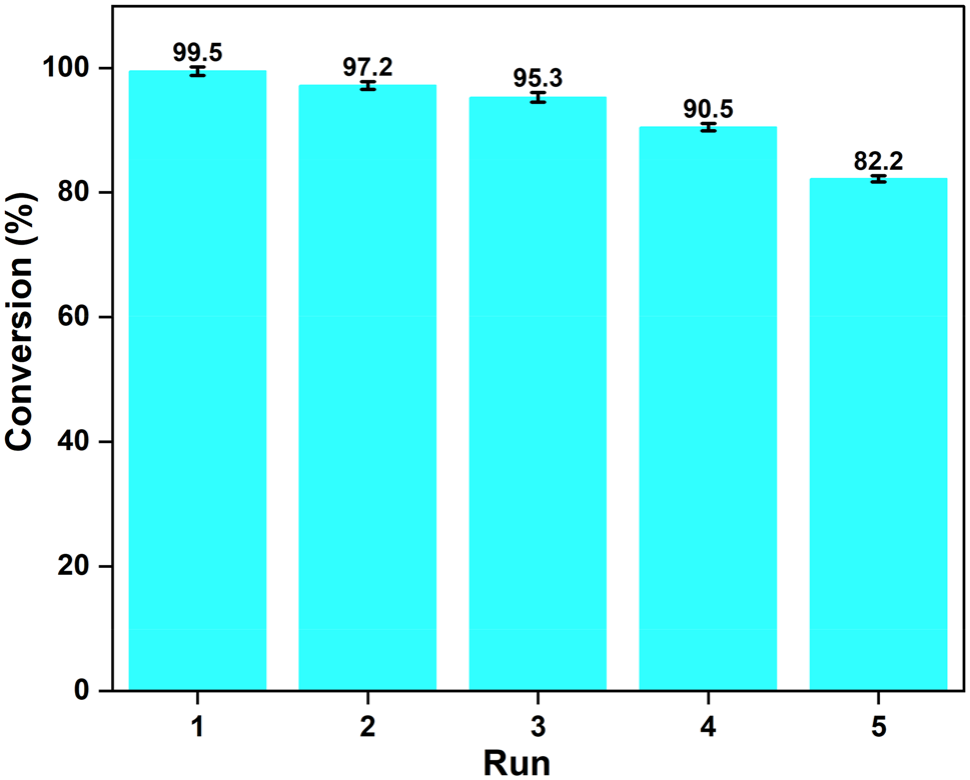
Graphical representation of reusability of BP-SO_3_H over 5 cycles in esterifying oleic acid (Columns 1–5).

A heterogeneity test for the BP-SO_3_H-15-18-100 catalyst was performed using the "hot filtration method (Sheldon's test)"^47^. The catalyst was separated by filtration under hot conditions after 35 min of reaction, and a yield of 85% was observed. Then, the reaction was continued for another 30 min with catalyst-free, where the yield of the product, 86.6%, was achieved (see SI, Fig. S6). This study confirmed that an insignificant amount of soluble active species in the filtrate could no longer increase catalytic activity. Hence, the catalyst displayed heterogeneous nature. One of the key benefits of employing a heterogeneous catalyst for biodiesel synthesis is that the catalyst may be reused, reducing biodiesel production costs significantly. Authors can anticipate the economic viability of biodiesel production at a commercial scale using reusability analyses. The synthesized banana peel catalyst was subjected to repeated catalytic cycles under optimal conditions (20:1 MOMR, 8 wt% catalyst loading, 80 °C, 55 min) to study its reusable nature. Each catalytic run was followed by filtration and methanol wash to recover the catalyst. The recovered catalyst was dried in a vacuum oven at 80 °C overnight before being used in subsequent cycles. OA yield to methyl ester progressively declined with every subsequent cycle, eventually reaching 80% in the fifth cycle (Fig. 8). Methylation of sulfonic acid groups^83^ and leaching^83,84^ were the major causes that contributed to the decline in oleic acid yield in each reaction cycle. SEM and EDX of the recovered catalyst (Fig. S7) were performed, and there was observed a decrease in the level of sulfur content from 4.62 wt% (1.4437 mmol g^−1^) of fresh catalyst to 3.08 wt% (0.9625 mmol g^−1^) of recovered catalyst.

### Comparison of the present catalyst with the reported catalyst for esterification of OA to biodiesel

Table 4 compares the efficacy of the catalyst BP-SO_3_H to other reported acid-functionalized esterification catalysts. A majority of these proved successful in converting OA to FAME (excluding entry 11); however, drawbacks include high MOMR (entries 8,11,12), high catalyst loading (entries 3,4,9,10), elevated temperature (1,4,7,8,9,11), and long reaction time (entries 1–9). The turnover frequency (TOF, Eq. S1) of most reported catalysts was lower than BP-SO_3_H-15-18-100, whose TOF was 0.047 mol g^−1^ h^−1^. Even though Fe_3_O_4_@ZIF-8/TiO_2_ achieved a higher TOF value, the requirement for high MOMR, the non-biogenic (not renewable or non-biodegradable) nature of the catalyst, and due to lower biodiesel yield, its appeal has been reduced.

**Table 4:** Comparison of the present catalyst with previously reported catalysts for esterification of OA to biodiesel.

Sl. no	Catalyst	Conditions^a^	TOF (mol g^−1^ h^−1^)	Yield (%)	Ref.
1	Aminophosphonic acid resin D418	14:1, 10.2, 115, 10	0.003	92.02	^85^
2	SO_3_H-HM-ZSM-5-3	18:1,5.2, 88, 10	0.006	92.45	^68^
3	SiW_12_ anchored to Hβ	20:1, 30, 60, 10	0.001	86.0	^86^
4	[HMIM]HSO_4_	15:1, 14, 110, 8	0.003	95.0	^63^
5	E-P400-2-SO_3_H	15:1, 5, 80, 5	0.013	95.5	^55^
6	Zr_1.0_Fe_1.5_-SA-SO_3_H	12:1, 9, 90, 4	0.009	99.5	^87^
7	FSS-IL	10:1, 10, 100, 4	0.008	93.5	^88^
8	HZSM-5	45:1, 10, 100, 4	0.007	83.0	^89^
9	C-SO_3_H	16:1, 17, 95, 4	0.005	99.9	^90^
10	Oil cake waste-SO_3_H	12:1, 20, 60, 2	0.008	94.0	^91^
11	Biochar	30:1, 5, 315, 3	0.011	48.0	^92^
12	Fe_3_O_4_@ZIF-8/TiO_2_	30:1, 6, 50, 1.25	0.043	93.0	^93^
13	BP-SO_3_H-15-18-100	20:1, 8, 80, 55^b^	0.047	97.9	Present study

^a^MeOH: OA molar ratio, catalyst loading (wt%), temperature (°C), time (h); ^b^Time (min).

## Conclusion

In this work, sulfonic acid functionalized banana (*Musa acuminata*) peel was synthesized and used as a novel heterogeneous catalyst for biodiesel production from oleic acid as a test substrate. Successful sulfonation of *M. acuminata* sample was confirmed by physicochemical characterization. A single peak at 170 eV in the XPS spectrum confirmed that the sulfur in the catalyst is mainly in the form of a sulfonic acid group. An RSM-CCD approach predicted a biodiesel yield of 98.2% under the optimized reaction conditions. Experimentally, microwave heating observed an actual biodiesel yield of 97.9 ± 0.7%. Based on the results of this study, the RSM-CCD approach can be used to forecast biodiesel synthesis under a range of conditions, allowing for subsequent synthesis to be more effective. The catalyst showed excellent stability on repeated reuse without much loss in its activity, where a high conversion of 82.2 ± 0.5% methyl oleate biodiesel was observed on the fifth catalytic cycle. The yield decreased mainly due to sulfur leaching from 4.62 wt% (fresh) to 3.08 wt% (5th recycled), which was detected by SEM–EDX analysis. Thus, compared to synthetic catalysts, which are toxic, unsustainable, expensive, and non-biodegradable. The current catalyst has a profound impact on environmental conservation.

## Supplementary Information

Supplementary Information.

## Data Availability

The datasets used and/or analyzed during the current study available from the corresponding author on reasonable request.
