# Supplementary material for: Microwave-assisted biodiesel production using bio-waste catalyst and process optimization using response surface methodology and kinetic study
Source: Sci Rep. 2023 Feb 13;13:2570. doi: 10.1038/s41598-023-29883-4 (PMC9925450; doi:10.1038/s41598-023-29883-4)
Supplement: Supplementary file 1 — Supplementary Information. [file 41598_2023_29883_MOESM1_ESM.docx]

**Supplementary Information**

**Microwave-assisted biodiesel production using bio-waste catalyst and process optimization using response surface methodology and kinetic study**

*Rhithuparna D^1^, Joseph VL Ruatpuia^1^, Shiva Prasad Gouda^1^, Pravin Kodgire^2, 3^, Sanjay Basumatary^4^, Gopinath Halder^5^, Samuel Lalthazuala Rokhum^1*^*

*^1^* Department of Chemistry, National Institute of Technology Silchar, Assam-788010, India

*^2^*Chemical Engineering Department, Pandit Deendayal Energy University, Gandhinagar-382426, Gujarat, India

*^3^*Center for Biofuel and Bioenergy Studies, Pandit Deendayal Energy University, Gandhinagar-382426, India

*^4^*Department of Chemistry, Bodoland University, Kokrajhar- 783370, Assam, India

*^5^*Department of Chemical Engineering, National Institute of Technology Durgapur, Durgapur 713209, West Bengal, India

Corresponding authors email: [*rokhum@che.nits.ac.in*](mailto:rokhum@che.nits.ac.in)

**Table of contents:**

| **Sl. No** | **Content** | **Figure** |
| --- | --- | --- |
|  | (a) XRD pattern, (b) TGA thermogram of BP and BP-SO_3_H-15-18-100, (c) N_2_ adsorption-desorption isotherm and BJH pore size distribution curve (inset) of the prepared sample BP-SO_3_H-15-18-100 and (d) FT-IR Spectra of BP (black-line), BP-SO_3_H-15-18-100 fresh catalyst (blue-line) and recovered catalyst (red-line). | Fig. S1 |
|  | SEM images of BP (a, b) (before sulfonation) and BP-SO_3_H-15-18-100 (c, d) (after sulfonation), along with TEM images of BP (e, f) and BP-SO_3_H-15-18-100 (g, h). | Fig. S2 |
|  | Elemental mapping unveiling carbon (a), oxygen (b), and sulfur (c) of sulfonated catalyst along with the SEM-EDX (d, e) before sulfonation and (f, g) after sulfonation. | Fig. S3 |
|  | (a) ^1^H NMR (500 MHz) and (b) ^13^C NMR (126 MHz) of methyl oleate biodiesel synthesized using BP-SO_3_H-15-18-100 catalyst. | Fig. S4 |
|  | GC of synthesized methyl oleate biodiesel | Fig. S5 |
|  | Heterogeneity test of BP-SO_3_H-15-18-100 catalyst. | Fig. S6 |
|  | SEM-EDX data of recovered catalyst. | Fig. S7 |
|  | Fuel properties of synthesized biodiesel | Table S1 |
|  | TOF calculation | *Eq. S1* |
|  | References | - |

**
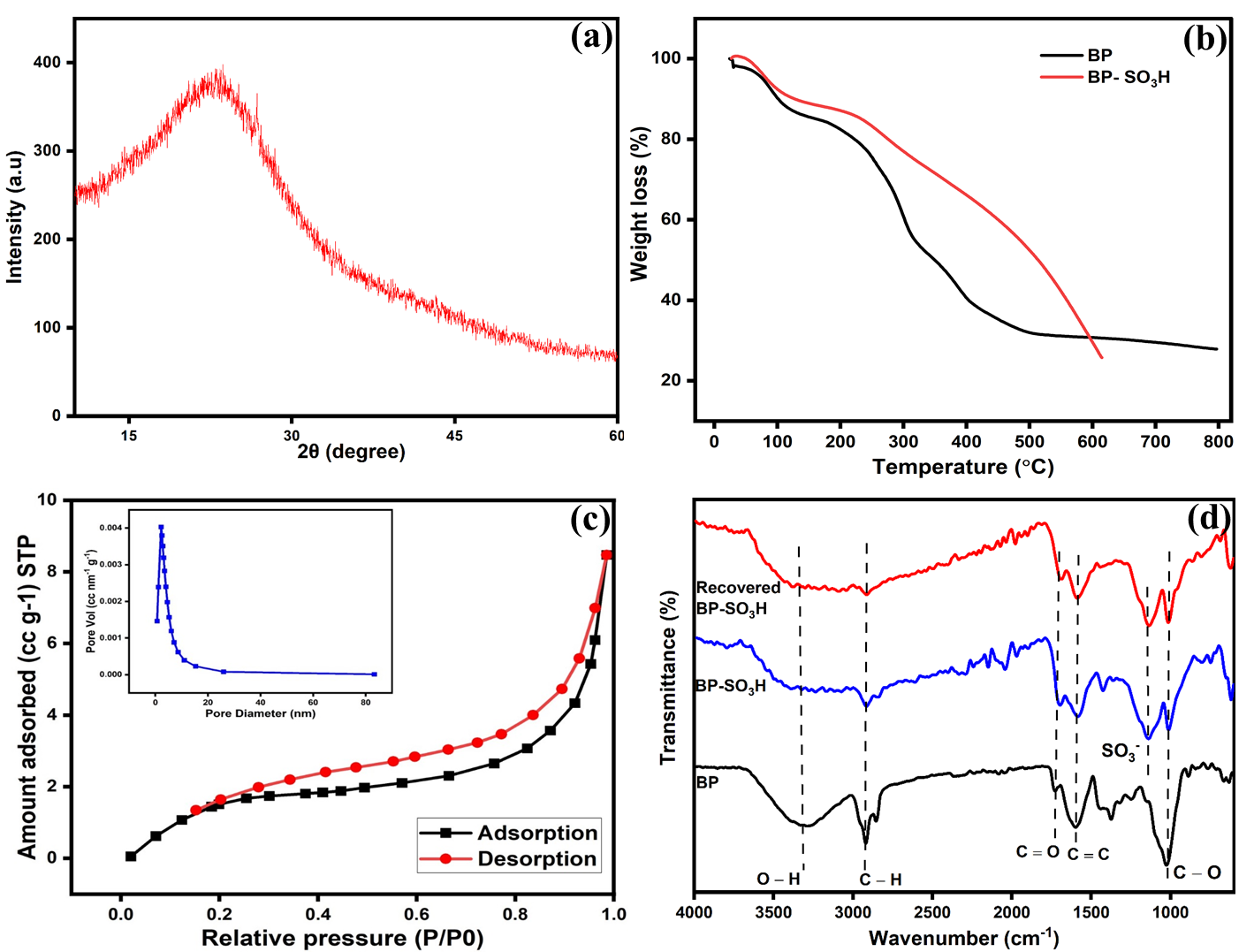
**

**Fig. S1:** (a) XRD pattern, (b) TGA thermogram of BP and BP-SO_3_H-15-18-100, (c) N_2_ adsorption-desorption isotherm and BJH pore size distribution curve (inset) of the prepared sample BP-SO_3_H-15-18-100 and (d) FT-IR Spectra of BP (black-line), BP-SO_3_H-15-18-100 fresh catalyst (blue-line) and recovered catalyst (red-line).

| 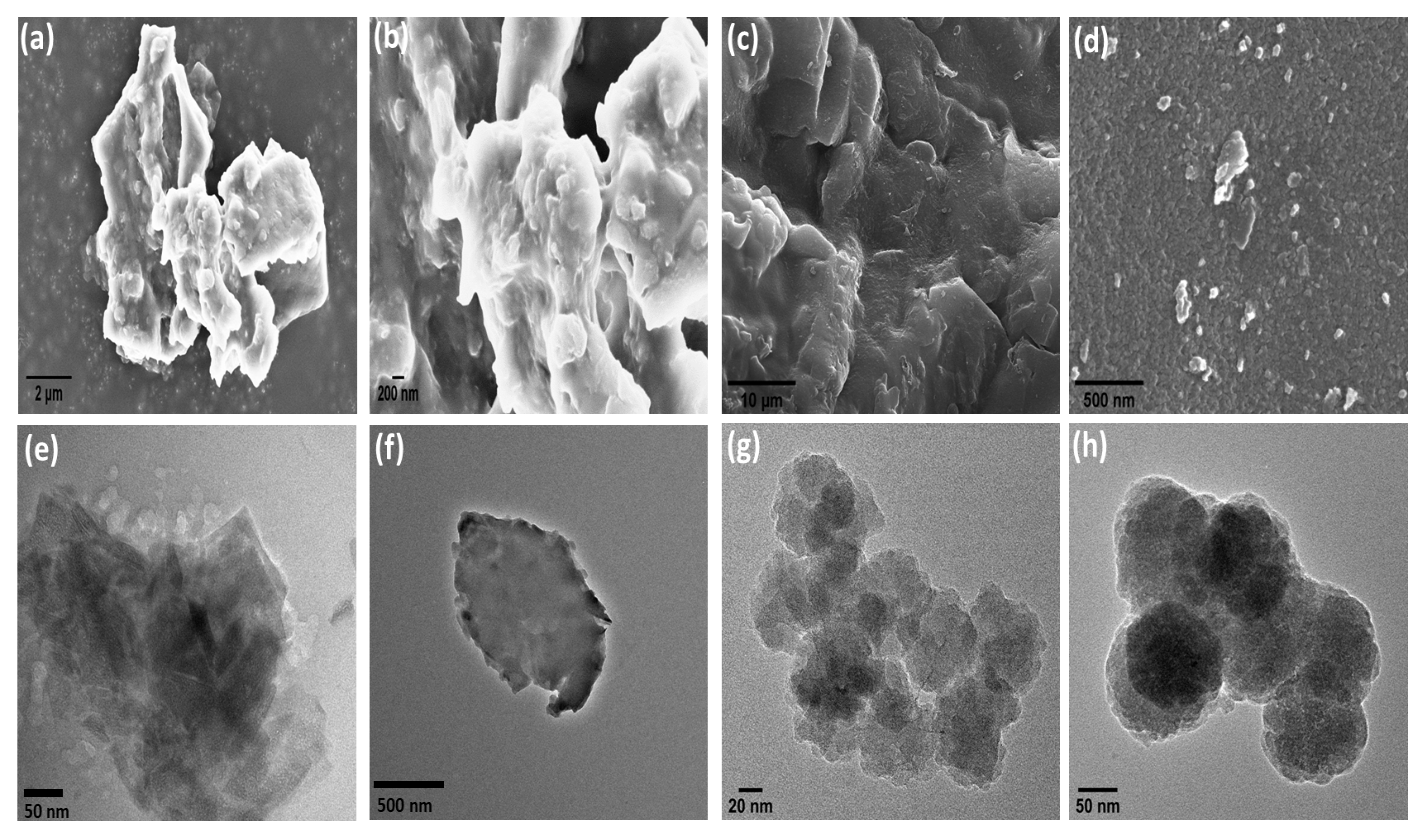 |
| --- |
| **Fig. S2:** SEM images of BP (a, b) (before sulfonation) and BP-SO_3_H-15-18-100 (c, d) (after sulfonation), along with TEM images of BP (e, f) and BP-SO_3_H-15-18-100 (g, h). |

| 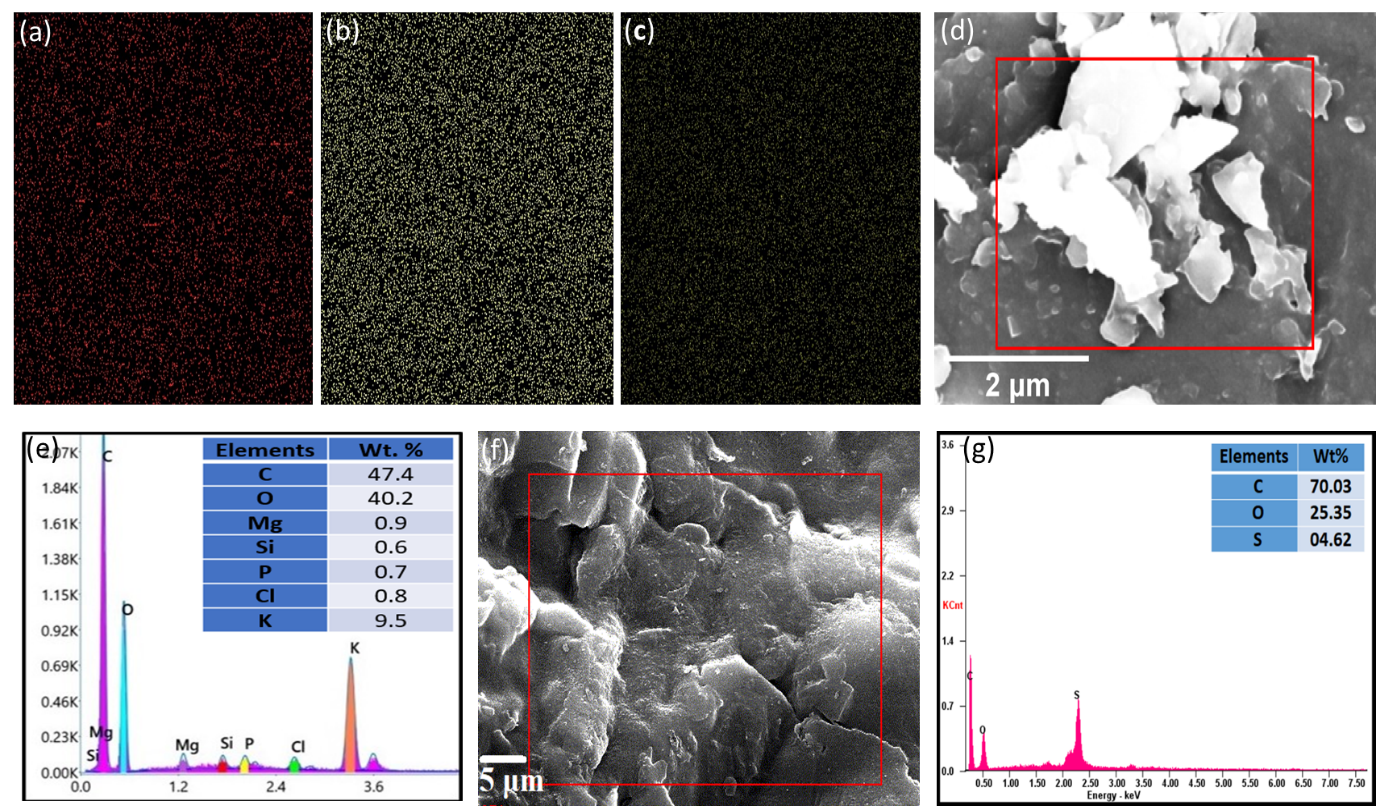 |
| --- |
| **Fig. S3:** Elemental mapping unveiling carbon (a), oxygen (b), and sulfur (c) of sulfonated catalyst along with the SEM-EDX (d, e) before sulfonation and (f, g) after sulfonation. |

| **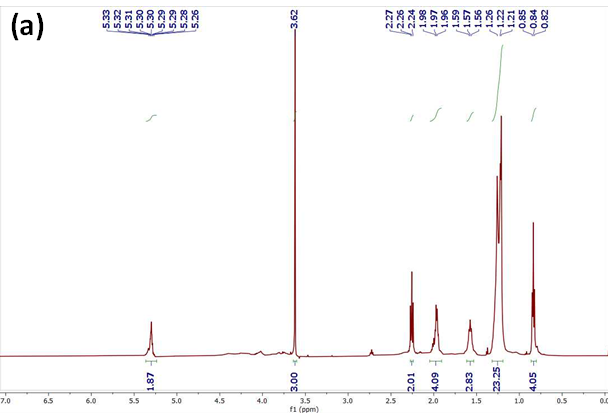** |
| --- |
| **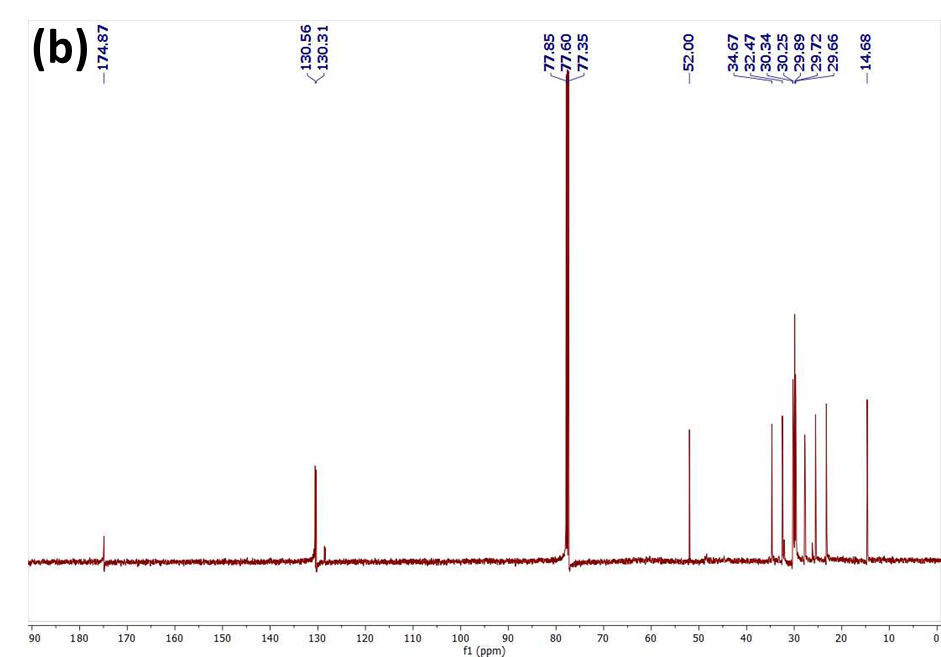** |
| **Fig. S4:** (a) ^1^H NMR (500 MHz) and (b) ^13^C NMR (126 MHz) of methyl oleate biodiesel synthesized using BP-SO_3_H-15-18-100 catalyst. |

NMR spectroscopic data for methyl oleate: **^1^H NMR** (500 MHz, CDCl_3_): 5.30 (m, 2H), 3.62 (s, 3H), 2.26 (t, 2H), 1.97 (m, 4H), 1.57 (m, 2H), 1.22 (m, 20H), 0.84 (t, 3H).**^13^C NMR** (126 MHz, CDCl_3_): 174.8, 130.5, 130.3, 52, 34.6, 32.4, 30.3, 30.2, 29.8-29.6, 14.6 ^1–4^.

| 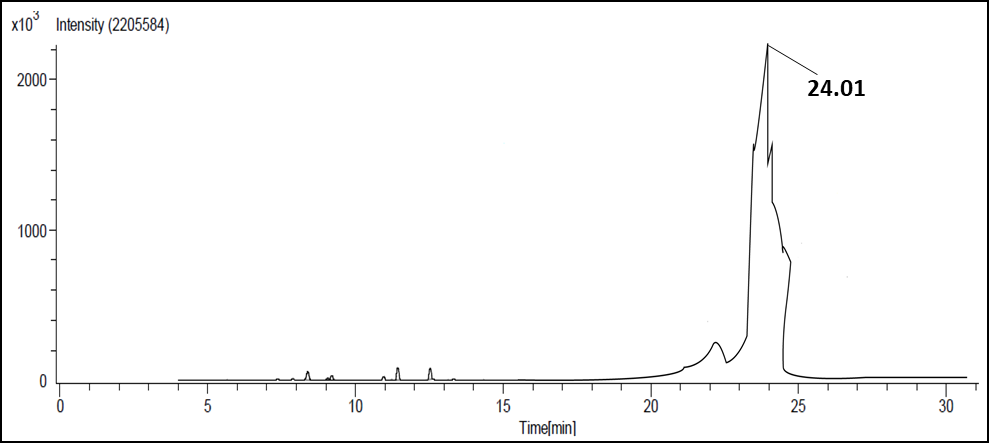 |
| --- |
| **Fig. S5:** GC of synthesized methyl oleate biodiesel. |

**Hot filtration method (Sheldon's test)**

**
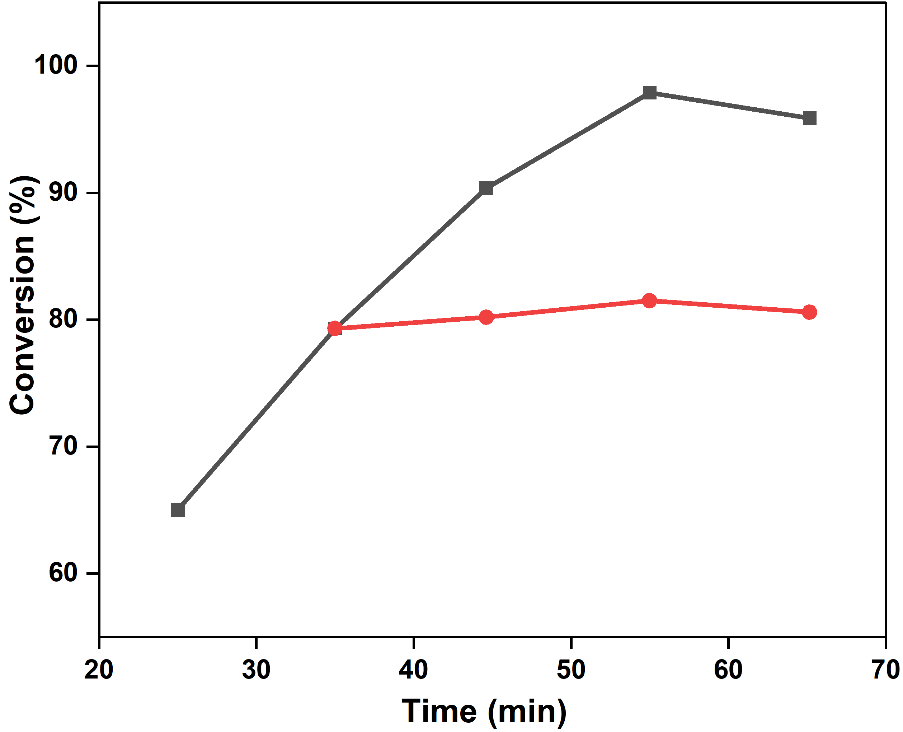
**

**Fig. S6:** Heterogeneity test of BP-SO_3_H-15-18-100 catalyst.

**
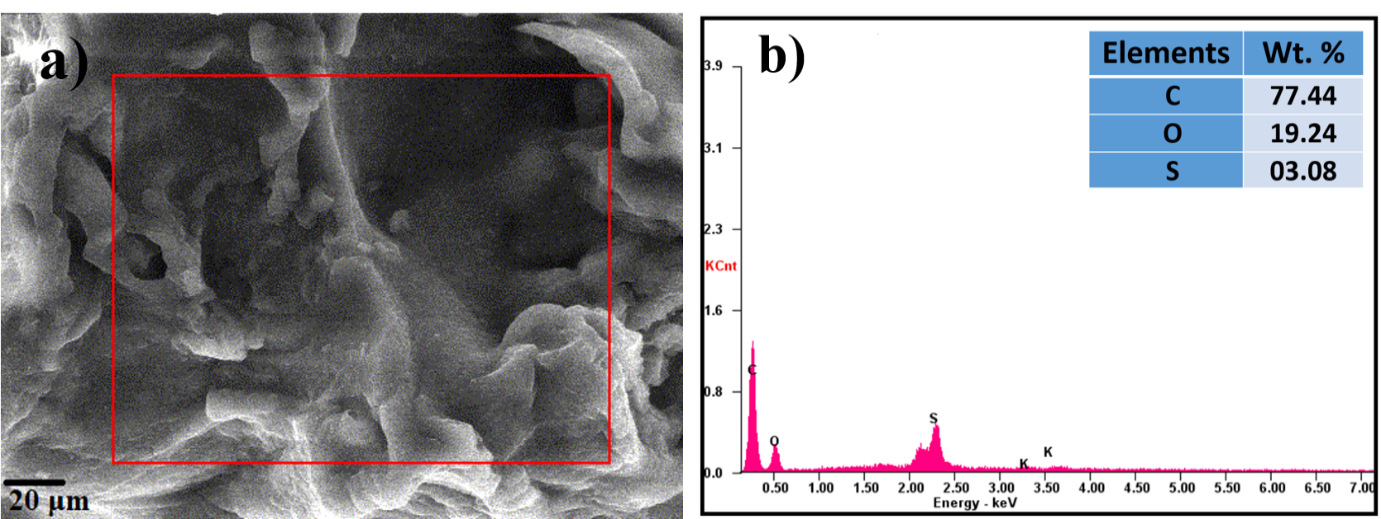
**

**Fig. S7:** SEM-EDX data of recovered catalyst.

**Fuel properties of synthesized biodiesel**

Using standardized ASTM (American Society for Testing and Materials) methods, the produced biodiesel was analyzed for its physical and chemical characteristics: density, kinematic viscosity, flash point, calorific value, and acid value (Table S1). Since every property lies in the range of ASTM standards, the synthesized biodiesel has potential to be used as an alternate fuel in diesel engines.

| **Table S1:** Comparison of physicochemical properties of produced biodiesel with ASTM-D6571 standards. | | |
| --- | --- | --- |
| **Properties** | **ASTM limit** | **Produced biodiesel** |
| Density (kg/m^3^) | 860-900 | 874 |
| Kinematic viscosity at 40 ℃ (mm^2^/s) | 1.9-6 | 4.52 |
| Flashpoint (℃) | 100-170 | 161 |
| Calorific value (MJ/kg) | 35-45 | 41.2 |
| Acid value | Max 0.5 | 0.1 |

**Turnover frequency calculation**

TOF = $\frac{\% Conversion oleic aicd}{(Time)(Catalyst wt.\%)(Molar mass oleic acid)} Eq. S1$

**References**

1. Rokhum, S. L., Changmai, B., Kress, T. & Wheatley, A. E. H. A one-pot route to tunable sugar-derived sulfonated carbon catalysts for sustainable production of biodiesel by fatty acid esterification. *Renew. Energy* **184**, 908–919 (2022).

2. Tariq, M. *et al.* Identification, FT-IR, NMR (^1^H and ^13^C) and GC/MS studies of fatty acid methyl esters in biodiesel from rocket seed oil. *Fuel Process. Technol.* **92**, 336–341 (2011).

3. Mello, V. M., Oliveira, F. C. C., Fraga, W. G., Do Nascimento, C. J. & Suareza, P. A. Z. Determination of the content of fatty acid methyl esters (FAME) in biodiesel samples obtained by esterification using ^1^H-NMR spectroscopy. *Magn. Reson. Chem.* **46**, 1051–1054 (2008).

4. Basumatary, S., Barua, P. & Deka, D. C. Identification of chemical composition of biodiesel from Tabernaemontana divaricata seed oil. *J. Chem. Pharm. Res.* **5**, 172–179 (2013).
